# Leveraging Citizen Science to Improve Urban Public Health

**DOI:** 10.1093/eurpub/ckac129.296

**Published:** 2022-10-25

**Authors:** J Hotting, T Clemens, R Guinto, M Commers, H Brand, K Czabanowska, RJJ Elands

**Affiliations:** 1 International Health, Maastricht University, Maastricht, Netherlands; 2 Geography, University of Sheffield, Sheffield, UK; 3 Historical Demography and Family History, Radboud University, Nijmegen, Netherlands; 4 Planetary and Global Health Program, St. Luke's Medical Center College of Medicine, Quezon City, Philippines; 5 Sunway Centre for Planetary Health, Sunway University, Selangor, Malaysia

## Abstract

**Background:**

Mobile health approaches in community based participatory projects keep the promise to encourage sustainable behavior in citizens by engaging communities in data collection and connecting their individual level behavior to larger level community health. This review evaluates the effectiveness of the implementation of citizen science for health promotion of non-communicable diseases in urban community-based participatory research projects.

**Methods:**

A scoping review was conducted using PRISMA-ScR. Pubmed and Web of Science were searched for citizen science studies. We included research 1.) conducted in an urban setting, 2.) related to environmental sustainability, 3.) focused on non-communicable diseases, and 4.) applied citizen science methodology.

**Results:**

32 community-based participatory research projects were identified using the following data collection technologies: mobile applications (n = 7), photovoice (n = 6), Stanford Healthy Neighborhood Discovery Tool (n = 6), monitoring (n = 6), mixed-methods (n = 4) and sensors (n = 3). The Stanford Healthy Neighborhood Discovery Tool was most effective at delivering real time data collection, exploring new communication and dissemination opportunities for remote and marginalized communities, and at offering a flexible and cost-effective approach to identify health promotion interventions. Studies that implemented photovoice tools and mobile applications had challenges with regard to recruitment and retention of participants as well as privacy concerns.

**Conclusions:**

Mobile health technologies in community-based participatory research projects may be a promising way to uncover unknown local risk factors, raise awareness and identifying targeted policy solutions to promote healthy and sustainable environments in urban spheres. The effectiveness of mobile health applications for health promotion of non-communicable diseases may vary between community-based participatory studies by data collection method.

**Key messages:**

• Mobile health technologies in communities may be a promising way to promote healthy and sustainable environments by raising awareness and offering targeted policy solutions in urban spheres.

• The effectiveness of mobile health applications for health promotion of non-communicable diseases may vary between community-based participatory studies by data collection method.

